# A general Bayesian treatment for MEG source reconstruction incorporating lead field uncertainty

**DOI:** 10.1016/j.neuroimage.2012.01.077

**Published:** 2012-04-02

**Authors:** J.D. López, W.D. Penny, J.J. Espinosa, G.R. Barnes

**Affiliations:** a Mechatronics School, Bl. M8-108 Facultad de Minas, Universidad Nacional de Colombia, Medellín, Colombia; b Wellcome Trust Centre for Neuroimaging, University College, London WC1N 3BG, UK

**Keywords:** MEG inverse problem, Co-registration, Metropolis algorithm, Bayesian model averaging

## Abstract

There is uncertainty introduced when a cortical surface based model derived from an anatomical MRI is used to reconstruct neural activity with MEG data. This is a specific case of a problem with uncertainty in parameters on which M/EEG lead fields depend non-linearly. Here we present a general mathematical treatment of any such problem with a particular focus on co-registration. We use a Metropolis search followed by Bayesian Model Averaging over multiple sparse prior source inversions with different headlocation/orientation parameters. Based on MEG data alone we can locate the cortex to within 4 mm at empirically realistic signal to noise ratios. We also show that this process gives improved posterior distributions on the estimated current distributions, and can be extended to make inference on the locations of local maxima by providing confidence intervals for each source.

## Highlights

► We show how to account for lead field uncertainty in the MEG inverse problem. ► Co-registration errors propagate through to the final current density estimate. ► It is possible to recover the location of the head based on MEG data alone.

## Introduction

The MEG inverse problem is ill-posed. One commonly used constraint to help solve this problem is to assume that the anatomy is known (Baillet & Garnero, 1997; Lin et al., 2006). The knowledge of anatomy, in the form of a cortical surface, coupled with the assumption that currents flow normal to this surface, considerably simplifies the problem. However, the accuracy to which the cortical surface can be known, given only scalp surface landmarks is finite. This anatomical information is often conveyed through three fiducial markers, sometimes markers plus a headshape, and sometimes in the form of bite-bar coordinates; the error implicit in all these approaches is of the order of 5 mm (Adjamian et al., 2004; Whalen et al., 2008). The degree to which these co-registration errors affect different algorithms is variable and has not been consistently studied. We know however that very small errors in mesh location for algorithms such as beamformers have been shown to be extremely detrimental (Hillebrand & Barnes, 2003, 2011). For example, so as not to degrade beamformer performance the cortical surface must be accurate to within 2 mm and the surface normals to within 10° (Hillebrand & Barnes, 2003). To our knowledge there is currently no method by which errors implicit in co-registration can be accounted for and propagated through to the source reconstruction stage.

The co-registration problem is just one example of a situation in which there is uncertainty on parameters on which the MEG lead fields depend non-linearly (for example the shape of the cortical mesh, the source extent, the model of the skull boundary). In this paper we outline a general mathematical framework which could be used to deal with any of these unknowns. As an example we demonstrate methodology to recover the location of the cortical sheet with only approximate (± 2 cm) prior knowledge of the head location. Implicit in this approach is an estimate of cortical activity that accounts for uncertainty in head position. Our approach combines deterministic and stochastic Bayesian inference procedures. Given an assumed head position we use the Multiple Sparse Priors (MSP) algorithm (Friston et al., 2008) to estimate sources, and to provide an estimate of the model evidence. This is combined with a stochastic search over head positions using a Metropolis approach (Gelman et al., 2000). Finally, proposed solutions are combined using Bayesian model averaging (BMA) (Trujillo-Barreto et al., 2004).

For each head position we used MSP with models comprising head location as free parameters and evaluated the free energy of the solutions over models. The model evidence embodies a compromise between model accuracy and complexity; if the head is placed in the wrong position the free energy will be decreased because a more complex model will be required to fit the data to the same accuracy. In the noiseless case, the model evidence will be maximised when the model parameters match the true surface position. However, system noise and local maxima in the fitting functions can give rise to a poorly defined error surface. We therefore use a Metropolis search, a Markov chain Monte Carlo (MCMC) strategy to search over head positions. We then use BMA to get an optimal model from those in the region of this global maximum. This approach allows us to also compute posterior distributions for current density, head location and peak source location that factor in both noise in the signal and noise in the co-registration procedure.

This paper is divided into the following main sections. We first present a brief explanation of the inverse problem formulation from a Bayesian perspective; this is followed by a description of the Metropolis algorithm used to search anatomical parameter space; we then describe how BMA is used to combine solutions over models based on different head positions. In summary, we show that it is possible to both effectively reconstruct brain activity whilst correctly accounting for anatomical (and signal) uncertainty, and also recover the location of the head based on MEG data alone.

## Theory

The magnitude of the magnetic fields observed over the scalp with MEG can be obtained from the quasi-static approximation of Maxwell equations and Poisson's equation (Hallez et al., 2007). This allows us to write the general linear model:

(1) $$Y=L_{a}J+\in$$

where $Y\in R^{N_{c}\times N_{t}}$ is the MEG dataset of *N*_*c*_ sensors and *N*_*t*_ time samples, $J\in R^{N_{d}\times N_{t}}$ the amplitude of *N*_*d*_ current dipoles distributed through the cortical surface with fixed orientation perpendicular to it, ∈ is zero mean Gaussian noise, and *L*_*a*_ is a gain matrix that embodies our assumptions about anatomy, *a*. This includes assumptions about head location, or the details of the particular cortical surface. We thereby write the likelihood associated with (1) to reflect this dependence:

(2) $$p(Y|J,a)=N(Y;L_{a}J,R)$$

with *N*(⋅) the multinormal density function and R the sensor noise covariance. Similarly, our prior assumption about source activity depends on anatomy

(3) $$p(J|a)=N(J;0,Q_{a})$$

where *Q*_*a*_ is the source covariance dependent on parameter set *a*. This leads to a posterior over sources via Bayes rule

(4) $$p(J|Y,a)=\frac{p(Y|J,a)p(J|a)}{p(Y|a)}$$

where the term in the denominator is known as the evidence. Eq. (4) makes explicit that source reconstructed solutions are dependent on anatomical assumptions. Anatomy may comprise multiple sets of parameters, e.g. *a* = {*h*, *w*, *s*}, where *h* denotes head location, *w* the spatial extent of cortical patches, or *s* the coefficients of a Fourier basis set describing the cortical surface.

The posterior over anatomical parameters is also, naturally, given by Bayes rule:

(5) $$p(a|Y)=\frac{p(Y|a)p(a)}{p(Y)}\text{.}$$

Importantly, the likelihood of anatomical parameters, *p*(*Y*|*a*), is equivalent to the evidence of the source reconstruction in Eq. (4).

In this paper we focus solely on head position: *a* ≡ *h*. Our prior assumptions about anatomy *p(h)*, can take many forms, here we use a uniform (flat) prior *p*(*h*) = *U*(*h*_0_, *σ*) for the head position, where *σ* represents the region where the head could be inside the MEG device.

Because the lead field *L*_*a*_, and therefore the likelihood *p*(*Y*|*J*, *a*), and source posterior *p*(*J*|*Y*, *a*) are highly non-linear functions of *a*, we propose to search anatomical space using a stochastic method such as the Metropolis algorithm (Gelman et al., 2000).

### Bayesian framework for the MEG inverse problem

For a given head model *h*_*k*_, the distributed source solution (*N*_*c*_ ≪ *N*_*d*_) is an ill-posed problem, because the lead field *L*_*h*_*k*__ in Eq. (1) is non invertible. To solve the problem it is necessary to find an inverse operator $M_{h_{k}}\in R^{N_{d}\times N_{c}}$:

(6) $$\hat{J}=M_{h_{k}}Y\text{.}$$

This problem can be solved in the Bayesian framework by assuming that *J* and *ε* are zero mean Gaussian:

p(J)=N(J;0,Q)p(∈)=N(∈;0,R).

Note that the distribution of the dipoles inside the head remains constant independently of the head location, but the prior source covariance matrix may be affected if it depends on the head model for its computation (*Q* would change to *Q*_*h*_*k*__). By applying the Gaussian probability distribution for a given head location *h*_*k*_, Eq. (4) leads to:

(7) $$p(J|Y,h_{k})\propto exp(-{\left(L_{h_{k}}J-Y\right)}^{\prime }R^{-1}(L_{h_{k}}J-Y)-J^{\prime }Q^{-1}J)$$

where (⋅)′ is the transpose operator. Given that the expected changes are small it is preferable to maximize the logarithm of the posterior: *log*(*p*(*J*|*Y*, *h*_*k*_)) ∝ *Ψ*, with:

(8) $$\Psi =-{\left(L_{h_{k}}J-Y\right)}^{\prime }R^{-1}(L_{h_{k}}J-Y)-J^{\prime }Q^{-1}J\text{.}$$

The estimated activity is obtained with $\hat{J}=argmax_{J}\{\Psi \}$ by differentiating Eq. (8):

(9) $$\frac{d\Psi }{dJ}{|}_{J=\hat{J}}=0=-2L_{h_{k}}^{'}R^{-1}(L_{h_{k}}\hat{J}-Y)-2Q^{-1}\hat{J}$$

which after some algebra leads to:

(10) $$\hat{J}=QL_{h_{k}}^{'}{\left(R+L_{h_{k}}QL_{h_{k}}^{'}\right)}^{-1}Y$$

which is the solution for reconstruction algorithms based on Gaussian assumptions (Grech et al., 2008). The posterior source covariance is given by

(11) $$\text{cov}(\hat{J})=\Sigma_{J}=Q-QL_{h_{k}}^{'}{\left(R+L_{h_{k}}QL_{h_{k}}^{'}\right)}^{-1}L_{h_{k}}Q$$

and can be used to provide error bars or confidence intervals on the solution. Both estimates of posterior mean and covariance require a known prior source covariance matrix *Q*, and known sensor noise *R*.

### Multiple Sparse Priors (MSP) algorithm

The accuracy of the reconstructed three dimensional map of source activity is highly dependent on the constraints *Q* and *R* used in Eqs. (10) and (11). We assume that the sensor noise covariance matrix *R* = *exp*(*λ*)*I*_*N*_*c*__, where $I_{N_{c}}\in R^{N_{c}\times N_{c}}$ is an identity matrix, and *exp*(*λ*) is the sensor noise variance. That is, the amount of noise variance is the same on all sensors (uniformity). The parameter *λ* can be positive or negative and is exponentiated to enforce positivity. This parameter can also be viewed as a regularization parameter or hyperparameter (Phillips et al., 2002b).

There are multiple constraints that can be used for the prior covariance *Q*. The simplest (minimum norm) assumption about the sources is that all the dipoles have approximately the same prior variance and no covariance: *Q* = *I*_*N*_*d*__. A more realistic assumption is a weighted sum of a set of *N*_*p*_ predefined covariance matrices *D* = {*D*_1_, …, *D*_*N*_*p*__}:

(12) $$Q=\sum_{i=1}^{N_{p}}\;exp(\lambda_{i})D_{i}$$

These matrices can be generated with prior information (such as fMRI data), or with a template like the Green's function based on a graph Laplacian (Harrison et al., 2007). Each of these *N*_*p*_ components defines a potential activated region of cortex,with hyperparameters *λ* = {*λ*_1_, …, *λ*_*N*_*p*__} pruned to those *D* corresponding to activated regions. This is the assumption underlying the MSP algorithm (Friston et al., 2008).

### Free energy as objective function

For the linear Gaussian models underlying MEG source reconstruction, the model evidence is well approximated by the negative variational free energy (henceforth “Free Energy”) (Friston et al., 2007; Penny, 2012; Wipf & Nagarajan, 2009). The free energy allows one to determine the most adequate model for a given dataset. For the model associated with a given head location *h*_*k*_ it can be expressed as a trade off between accuracy and complexity:

(13) $$F(h_{k})=Accuracy(h_{k})-Complexity(h_{k})$$

where the accuracy depends on the estimation error $e_{Y}=Y-M_{h_{k}}\hat{J}$, the model based sample covariance matrix: *C*_*Y*_ = *R* + *LQL*′, and the number of samples available *N*_*t*_:

(14) $$Accuracy(h_{k})=-\frac{1}{2}e_{Y}^{'}C_{Y}^{-1}e_{Y}-\frac{1}{2}log|C_{Y}|-\frac{N_{t}}{2}log2\pi$$

with | ⋅ | the matrix determinant operator. When searching for the optimal head position the MEG data do not change, so *N*_*t*_ is the same for all the models; and the accuracy is affected by the estimation error *e*_*Y*_, and the model covariance *C*_*Y*_, decreasing the free energy for inaccurate models.

In the MSP algorithm the complexity only depends on the hyperparameters *λ*, that control the power allocated to each of the source components. Here we consider the prior *p*(*λ*) and approximate posterior *q*(*λ*) densities of the hyperparameters as Gaussian distributed:

$$p(\lambda )=N(\lambda ;\nu ,C_{\lambda })q(\lambda )=N(\lambda ;\mu ,\Sigma_{\lambda })\text{.}$$

For the head location *h*_*k*_, the complexity of the solution is defined as:

(15) $$Complexity(h_{k})=\frac{1}{2}e_{\lambda }^{'}C_{\lambda }^{-1}e_{\lambda }+\frac{1}{2}log\frac{\left|C_{\lambda }\right|}{\left|\Sigma_{\lambda }\right|}$$

where the error *e*_*λ*_ = *μ* − *ν* and the posterior covariance of the hyperparameters *Σ*_*λ*_, are calculated within the MSP estimation. In absence of prior information the initial values of the hyperparameters can be considered uninformative: *ν* = 0, and their prior variances: *C*_*λ*_ = *αI*_*N*_*p*__, with *α* large. Based on the definition of complexity, it can be concluded that the use of a large number of hyperparameters increases the complexity and reduces the freeenergy.

In this work the free energy is used in two different ways. First, it provides an objective function for hyperparameter optimization. The optimal set of hyperparameters for a given head location *h*_*k*_ is achieved with the maximum free energy value: $\hat{\lambda }=arg\;max_{\lambda }F(h_{k})$. Second, because free energy approximates the log model evidence it can be used to score source reconstructions based on different head locations. That is, the reconstruction of each head location has an associated free energy that can be compared with those of other head locations, in order to find the optimum.

### Search algorithm

**Fig. 1:**
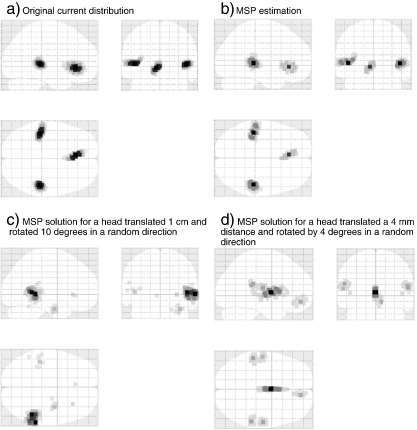
Glass brain with simulated brain activity: (a) Original brain map, (b) MSP estimate of the simulated data. (c) and (d) Examples of different MSP reconstructions with co-registration error; on (c) one lateral source keeps most of the energy and the other two almost disappear, on (d) a ghost source appears in the middle of the brain.

**Fig. 2:**
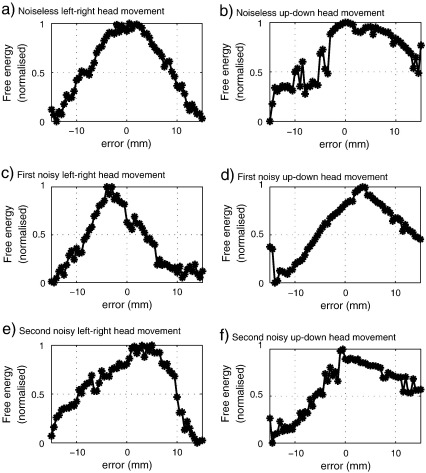
Graphs of different normalised free energy trajectories for a single axis with 0.5 mm of resolution: (a) presents the movement on the left–right axis. (c) and (e) show the same left–right movement using data with *SNR* = 0 dB, both the waveform and the maxima varied. (c) shows the up–down movement, note that the free energy seems to be higher when the head is near the sensors. (d) and (f) show the same up–down movement using noisy data with *SNR* = 0 dB, again the free energy trajectory varies for each case.

In Section Free energy as objective function we stated that each head location has an associated Free energy. These Free energy values are approximate the log model evidence: *F* ≈ *logp*(*Y*|*h*). Applying the free energy criterion to the models which vary only in head position keeps the data and the number of parameters unchanged, but it affects the complexity. One would expect that the maximum free energy corresponds to the true position of the head, because any other location would require more complexity to explain the same data with the same accuracy (See Figs. 1 and 2 and their discussion).

For a single dataset the Free energy is a function of head position and orientation, which implies six degrees of freedom (three for position and three for orientation). The head location *h*_*k*_ is specified by three fiducials in MEG sensor space: Nasion, left ear, and right ear. Movement between head locations *h*_0_ and *h*_1_ is performed by a rigid body transformation over the three fiducials (Friston et al., 2006, chap. 4). Here the mid-point between the left and right ear fiducials is used as the origin of rotation of the head.

In practice, there is always some uncertainty about the head location inside the helmet, but for the purposes of this demonstration we will consider the worst case: a uniform probability distribution of the head location *p(h)* inside the search space, which in this case is the space inside the MEG helmet allowing free rotation of the head.

The problem is now how to search this space. One possibility would be to create a grid and evaluate *F* at each position. This is however too computationally demanding for a six-dimensional space. Another possibility would be a deterministic algorithm which follows the gradients of *F* with respect to head position. Given that this search space is highly non-linear (see later) this would be suboptimal. For these reasons we have chosen to use the Metropolis search strategy. It consists of following a Markov chain with variable step given by a probability distribution centred on the last step. Parameters are updated so as to follow increasing *F* values, but decreases are also allowed (in order to avoid getting stuck in local extrema).

#### Metropolis search

The Metropolis search algorithm is part of a family of MCMC techniques (Gelman et al., 2000) that allows several problem specific modifications. Here we describe the algorithm implemented to search for the true head location:

1. Select a random sample from the prior over head location: *h*_0_ ∼ *p*(*h*), solve the MSP reconstruction for that location and calculate the corresponding free energy value *F*(*h*_0_).
2. Use a Gaussian proposal distribution to obtain a new set of fiducials near to the head location computed on the previous step *h*_*k* − 1 _: $h^{\prime }∼N(h^{\prime };h_{k-1},\sigma^{2}I)$. For each of the six degrees of freedom we used the same standard deviation of $\sigma =2.4/\sqrt{6}$. With *σ* expressed in degrees for rotation, and *mm* for translation (Gelman et al., 2000). The parameter *σ* could be adjusted to improve the rate of convergence (Woolrich et al., 2006), but this is beyond the scope of the current paper.
3. Perform MSP reconstruction on the new location of the head and calculate the ratio with the new Free energy value *F*(*h*′):
  $$\begin{array}{c} r=\frac{p(Y|h^{\prime })p(h^{\prime })}{p(Y|h_{k-1})p(h_{k-1})}\\ =exp(F(h^{\prime })-F(h_{k-1}))\frac{p(h^{\prime })}{p(h_{k-1})} \end{array}$$
  the ratio is given by the comparison of log evidence between the previous reconstruction *p*(*Y*|*h*_*k* − 1 _), and the proposed one *p*(*Y*|*h*′), where each is also weighted by the prior. A ratio larger than one means that the proposed head location *h*′ has more evidence than the previous one.
4. Take a decision: If *r* > 1 then the new value is higher and accepted (*h*_*k*_ = *h*′); if *r* < 1, then the new value is compared with a random sample from a uniform distribution: *β* ∼ *U*(0, 1), if *r* > *β* it is accepted, or rejected otherwise: *h*_*k*_ = *h*_*k* − 1 _. Allowing transitions to lower probability values enables the algorithm to escape from local maxima.
5. Return to the second step and repeat until convergence. After an initial burn-in period, the samples *h*′ together comprise an approximate posterior distribution over the head locations *p*(*h*|*Y*).

The following section describes a standard method for determining when the sampling procedure has converged. After convergence, the first half of samples constitute a burn-in period and are discarded. This avoids dependence on the initial head position *h*_0_ of the sampling chain.

#### Use of convergence rule

The Metropolis algorithm presented above follows a single Markov chain to generate samples from the posterior *p*(*h*|*Y*), but it may fall into a local maxima or take an excessive amount of time to approximate the true posterior. In order to avoid these problems we use multiple chains simultaneously and define convergence based on the variance-between and -within chains (Gelman et al., 2000), i.e. each chain forms an approximate posterior *p*_*g*_(*h*|*Y*), for *g* = 1, …, *G* chains; then the variance within each chain and between chains is computed in order to determine convergence.

In brief, start *G* chains from different head locations. Then perform the Metropolis algorithm individually for each chain. Wait until all chains have a representative number of samples (here we used 100), then after each iteration of all chains, use the second half of samples (length *n*) to calculate $\sqrt{\hat{R}}$ for each of the selected scalar estimands (in this case we use *F*), and the algorithm finishes when $\sqrt{\hat{R}}\approx 1$. The parameter $\sqrt{\hat{R}}$ gives a tolerance limit for the Free energy variation.

The stopping parameter $\sqrt{\hat{R}}$, relates the marginal posterior variance *var*(*F*|*Y*) and the within-sequence variance *Z* for the scalar estimand *F*

(16) $$\sqrt{\hat{R}}=\sqrt{\frac{var(F|Y)}{Z}}$$

The marginal posterior variance *var*(*F*|*Y*) is computed as the weighted average of *Z* and the between-sequence variance *B*

(17) $$var(F|Y)=\frac{n-1}{n}Z+\frac{1}{n}B$$

with

(18) $$B=\frac{n}{G-1}\sum_{g=1}^{G}\;{\left({\bar{F}}_{\cdot g}-{\bar{F}}_{\cdot \cdot }\right)}^{2}$$

where ${\bar{F}}_{\cdot g}$ is the mean Free energy of the *g*-th sequence, and ${\bar{F}}_{\cdot \cdot }$ is the mean Free energy among sequences. The within-sequence variance is calculated as:

(19) $$Z=\frac{1}{G}\sum_{g=1}^{G}\;\left(\frac{1}{n-1}\sum_{i=1}^{n}\;{\left(F_{\mathrm{ig}}-{\bar{F}}_{\cdot g}\right)}^{2}\right)\text{.}$$

The quantity $\sqrt{\hat{R}}$ compares the variance of each independent Markov chain with the marginal posterior variance of all chains. If $\sqrt{\hat{R}}$ approaches unity then all chains should be sampling from the same density. This will occur when the chains have forgotten their initial states and have converged (Gelman et al., 2000).

### Bayesian model averaging

Our framework decouples inference about functionality, based on *p*(*J*|*Y*), from inference about anatomy *p*(*h*|*Y*). This allows us to use established algorithms to compute them: Inference about anatomy can be made solely on the posterior samples *h*_*k*_ obtained with the Metropolis algorithm, and inference about functionality is given by Bayesian model averaging

(20) $$p(J|Y)=\sum_{k}\;p(J|Y,h_{k})p(h_{k}|Y)$$

where *p*(*J*|*Y*, *h*_*k*_) is the distribution of the sources obtained with model *h*_*k*_. This is evaluated using

(21) $$p(J|Y)\approx \sum_{s}\;p(J|Y,h_{s})$$

where *h*_*s*_ are the posterior samples produced by the Metropolis algorithm. Whilst this equation can be used to compute a full posterior distribution over sources we are typically only interested in the posterior mean, $\hat{J}$. The following algorithm is used to provide an estimate of $\hat{J}$ and is used with *T* = 10, 000 iterations.

For t = 1,…,T do
  - a) Pick a head location from its posterior probability distribution: *h*_*k*_ ∼ *p*(*h*|*Y*)
  - b) For the head location *h*_*k*_ obtain the estimated values $\hat{J_{k}}$ and their posterior covariance *Σ*_*k*_, using Eqs. (10) and (11).
  - c) Obtain a normal random variable with mean ${\hat{J}}_{k}$ and covariance (*Σ*_*J*_)_*k*_: ${\tilde{J}}_{t}∼N({\tilde{J}}_{t}|{\hat{J}}_{k},{\left(\Sigma_{J}\right)}_{k})$. In practice, for computational efficiency and storage limitations only the main diagonal of each (*Σ*_*J*_)_*k*_ is computed in Eq. (11).
end for
- Obtain the mean of the random variables: $\hat{J}=\sum_{t}\;{\tilde{J}}_{t}$

In brief, step (a) renders the source estimate $\tilde{J}$ dependent on anatomical uncertainty and step (c) renders it dependent on measurement error. One can also use the *J*_*t*_ to produce confidence intervals over the location of the global maximum, or the maximum within a certain cortical region. Note that BMA is computationally very efficient, on a desktop computer it takes less than one minute.

## Results

In this section we first generate simulated MEG data for several source distributions and head locations and then attempt to estimate the head position and cortical current distribution under various noise conditions using the algorithm outlined above. We then also test our algorithm with previously published experimental data (Sedley et al., 2012).

### Description of the simulated data

Several single trial datasets of *N*_*t*_ = 161 samples over *N*_*c*_ = 274 MEG sensors were generated by projecting different source distributions into sensor space. Fig. 1(a) shows for example two synchronous lateral sources of neural activity with sinusoidal signals of 20 Hz, and a frontal source with sinusoidal signal of 10 Hz. Tests were performed noiseless and using *SNR* = {− 20, − 10, 0, 5, 10, 15, 20} decibels, with *SNR* = *log10*|var(*Y*)/var(*noise*)|. All sources were spatially normally distributed with full width half maximum of approximately 10 mm. The MSP algorithm was implemented over a mesh of *N*_*d*_ = 8, 196 dipoles distributed over the cortical surface, each with fixed orientation perpendicular to it; following the procedure proposed in (Phillips et al., 2002a).

The priors used to form the set of covariance components *D* of (12) were the same as implemented with the MSP algorithm in the Statistical Parametric Mapping (SPM8) software package.^1^ They consist of 512 covariance components with selected columns of a Green's function (Harrison et al., 2007) covering the entire cortical surface. The settings of the algorithm are also explained in (Friston et al., 2008). The translucent glass brains of Fig. 1 show the frontal, lateral and superior views of the 512 dipoles with highest variance during the time windows of interest.

For all tests we used a reduced model of the sensor space using the 100 largest eigenmodes, obtained with the singular value decomposition of the gain matrix and projecting the sensors into this new space (as explained in (Friston et al., 2008; Phillips et al., 2002a)), considerably reducing computation time. This dimension reduction was based only on the first head location lead field *L*_*h*_0__, and its decomposition was used in all the following iterations to guarantee the data did not change. In future it might be worth considering schemes in which this dimension reduction stage is removed once the approximate location of the global maximum is reached.

Fig. 1(b) shows the MSP solution for the true position of the head with the source distribution of Fig. 1(a). For the *i*-th source of neural activity, the estimation error was defined as the Euclidean distance between its location *S*_(*true*)_(*i*), and the location of the dipole with maximum energy after MSP reconstruction in the region near to the *i*-th original source, *S*_(*msp*)_(*i*):

(22) $$Error(i)=||S_{\left(msp\right)}(i)-S_{\left(true\right)}(i)||$$

The average source location error for the three source simulation, given the true head location, was zero (between Figs. 1(a) and (b)). This is not so surprising as the simulated neural sources were randomly placed at MSP patch centres. Note that we used a coordinate system for the dipoles referenced to the head itself, but we used a coordinate system referenced to MEG helmet to describe fiducial locations.

For the first validation of the proposed algorithm, the head was allowed to move 20 mm in each direction with the constraint of avoiding collision with the sensors. The orientation of the head was free to vary and it was always initialised in a random location within ± 15° from the true location. Two examples of the MSP reconstruction for a displaced head are presented in Figs. 1(c) and (d), and show how poor knowledge of the head location affects source reconstruction, both solutions have lower free energy than that at the true location. Note however that both these incorrect solutions arise from relatively small (and typical) co-registration errors (4 mm and 4° in 1(d)).

Our objective is to recover the true distribution of currents that generate the data, as well as the true head position. All the error measurements of sources of neural activity were made with respect to the original simulated distribution. The co-registration, forward problem (Nolte, 2003) and MSP inverse solution were obtained with the SPM8 software package.

### Illustrative example: single axis movement

For a better understanding of the different steps involved, we first explore head movement along a single dimension. Several tests were performed by allowing the head to move only on a single axis or orientation. This allowed us to compare the Metropolis algorithm with a simple grid search.

Fig. 2 shows the normalised (with respect to the maximal) free energy trajectories in six different realisations, the head was moved between ± 15 mm from its original position, 0.5 mm at a time. Fig. 2(a) shows a left–right movement, the positive values correspond to the right part of the head being nearer to the sensors. Fig. 2(b) shows an up–down movement, the upward movement corresponds to positive error values. There were higher free energy values when the head was nearer to the sensors. Figs. 2(c) and (e) show two different noisy (*SNR* = 0 dB) left–right head movement realisations, note that the peak free energies do not give zero error and are different to one another for the same underlying head location. The same situation can be seen in Figs. 2(d) and (f), where up-down noisy (*SNR* = 0 dB) realisations again present different solutions for the same head locations; this is an important motivation for the steps that follow.

The solution of the MSP for several head locations makes it necessary to generate a new lead field matrix at each iteration and then solve the MSP reconstruction, causing a high computational load. For an Intel Core i7 desktop computer with 6 GB of RAM memory each lead field matrix takes 30 s and the MSP solution takes approximately 80 s, using Matlab 2010b and multiprocessing functions; the simulations of Fig. 2(a) took approximately two hours. This means that the grid search presented in Fig. 2 is not computationally feasible for a problem with six degrees of freedom.

**Fig. 3:**
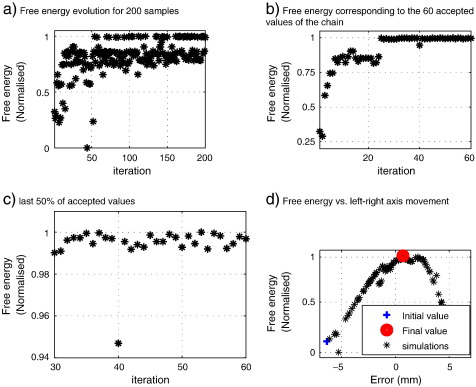
Metropolis search behaviour on a single axis with *SNR* = 0 dB. (a) Evolution of the free energy through the 200 samples. (b) Accepted values for step updating. (c) Second half of the chain, note that all of them remain near to the maximum. (d) Evolution of the Nasion fiducial position through the simulations, the starting value was in error by 6.3 mm and dropped to 0.7 mm after optimisation.

The Metropolis search was implemented to step through parameter space in the left–right axis with *SNR* = 0 dB. This algorithm is faster than a grid search and it allows one to avoid local maxima. Fig. 3(a) shows the normalised free energy update through 200 simulations; Fig. 3(b) shows 60 normalised free energy values accepted from the 200 simulations through the Metropolis iterative process, and the second half of samples used to form the posterior distribution *p*(*h*|*Y*) are shown in Fig. 3(c).

Fig. 3(d) shows the trajectory of the nasion fiducial position (originally at 0 mm). The posterior mean head position is shown in Fig. 3(d) (‘final value’) and is 0.7 mm from the true position.

**Fig. 4:**
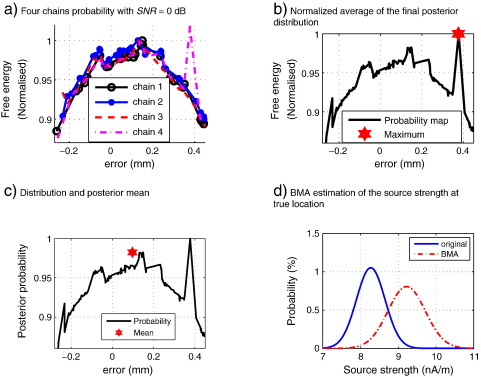
(a) normalised posterior probability distribution of four chains of simulations for a single axis problem (left–right) with *SNR* = 0 dB, note the erroneous global maximum in one of the chains. (b) normalised posterior distribution for the average of the four chains, the erroneous maximum remains as global maximum. (c) even though the posterior distribution contains the unwanted maximum, the mean is more robust and closer to the truth. (d) BMA provides the posterior distributions of the current density at each location; note that after allowing for co-registration error the distribution has widened slightly.

The Metropolis search algorithm proposed in Section Search algorithm is designed to sample from the posterior distribution over head locations *p*(*h*|*Y*), based on the convergence of multiple chains. Samples from the second half of all *G* = 4 chains were used to form this posterior density. The mean of this posterior distribution provides a robust estimate of head location whereas the maximum does not, as illustrated in Fig. 4 .

Fig. 4(a) shows the normalised free energy accounting 95% of cumulative probability distribution of four chains of 200 samples each, for a one degree of freedom movement of the head (left–right) with *SNR* = 0 dB, the fourth chain has a global maximum at around 0.4 mm. Fig. 4(b) highlights the maximum posterior value whereas Fig. 4(c) highlights the posterior mean, the latter being much closer to the true value.

An advantage of our inference framework is that we can obtain a posterior distribution of activity in each source location that takes into account uncertainty about anatomy, in this case head position. Similar inferences can be made about locations of peak activity (see later). Fig. 4(d) shows how the posterior source distribution changes (a change in the mean and increase in the variance) when the uncertainty in head location is taken into account (see Section Bayesian model averaging for methods).

### Simulation results

For the six degrees of freedom simulations the head was allowed to move by 20 mm in each direction with free rotation (360°) around all axes. Several tests were performed with noiseless and noisy data.

**Fig. 5:**
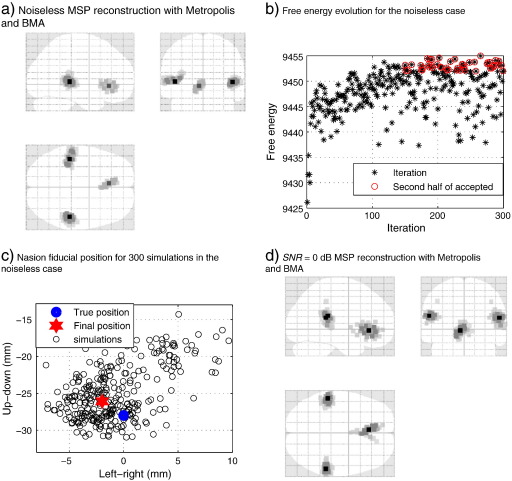
True head position estimation for: (a) Noiseless case, and (d) noisy *SNR* = 0 dB case; the algorithm manages to recover the true distribution (without ghost or missing sources) despite the noise. (b) shows the evolution of the Metropolis target distribution and the accepted values that were used in the BMA step. (c) shows the multiple positions of the Nasion fiducial tested by the search algorithm, with the posterior mean indicated by the final position.

Fig. 5(a) shows the estimated MSP image after applying the Metropolis and BMA algorithms, the average estimation error computed with Eq. (22) for the noiseless case is zero, and the FID positions had mean error of 2.2 mm. Fig. 5(b) shows the free energy evolution and the accepted values of the chain, and Fig. 5(c) shows the Nasion fiducial movements for the 300 samples in the *X-Z* axis (left–right–up–down), the error for this fiducial was 2.2 mm, but the search space was up to 20 mm per axis. That is, the prior over head location is a uniform density with width *σ* = 20 mm (see beginning of Section Theory). Fig. 5(d) shows the MSP reconstruction with Metropolis search and BMA for *SNR* = 0 dB, the three sources of neural activity were recovered with an average error of approximately 5 mm.

**Fig. 6:**
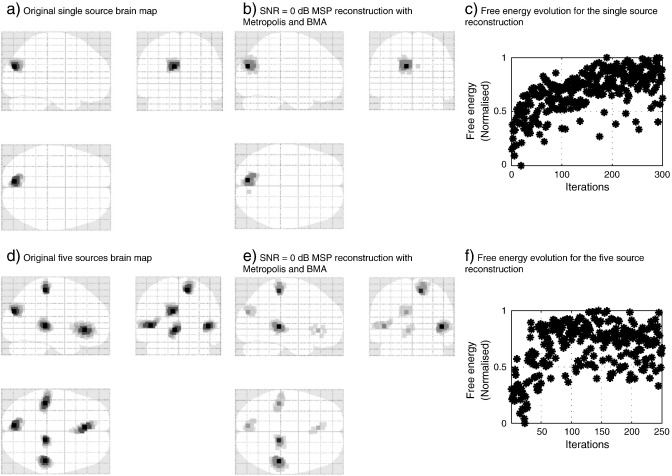
Example of MSP reconstruction with one and five synthetic sources. (a) The single simulated source. (b) MSP reconstruction after recovering the head location with Metropolis and BMA algorithms. (c) Normalised free energy over Metropolis iterations. (d) The distribution of the five simulated sources. (e) MSP reconstruction after recovering the head location. (e) Normalised free energy evolution through the Metropolis algorithm, generally the approximate posterior distribution converged in less iterations for the five source than for the single source simulations.

Fig. 6 shows the MSP reconstructed brain images for single and five source simulations. Figs. 6(a) and (d) show the original simulated current distributions for one and five sources respectively, Figs. 6(b) and (e) show their MSP reconstructions, and Figs. 6(c) and (f) show the normalised free energy evolution of the Metropolis algorithm for each case. In both cases the reconstruction error is approximately zero (≈ 0.5 mm).

**Fig. 7:**
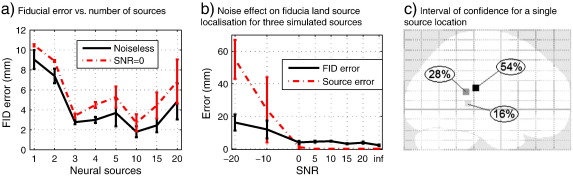
(a) Fiducial error for different source distributions in noiseless and *SNR* = 0 dB conditions; note that it becomes easier to define the head location when more sources are active, but this plateaus at around 4 mm and has little dependence on SNR. (b) Effect of noise for the three source simulation. At low SNR the head location is bounded by the prior uncertainty (20 mm) consequently source localisation error is even larger. Above 0 dB SNR however, the FID localisation error plateaus at around 4 mm; likewise the error in source localisation falls to zero. This plateau in fiducial error and unrealistically good source localisation performance are due to the fact that in the MSP sources are modelled as discrete non-overlapping patches and this leads to these quantisation effects. (c) An example of the confidence interval it is possible to place on peak source location (in this case for a single simulated source). This confidence interval takes account of both measurement and co-registration noise (in this case around 6 mm).

Fig. 7 shows a summary of the algorithm performance for different source configurations and noise conditions. For each current distribution or noise realisation, eight noisy datasets were generated and a set of *G* = 4 chains with different seeds were used to determine convergence. The Metropolis chains stopped after approximately 300 iterations in most cases (relatively modest number considering we are optimizing a function with six degrees of freedom).

Fig. 7(a) shows the fiducial error as a function of number of sources in the noiseless and *SNR* = 0 dB cases. For low number of sources (< 3) there is relatively little information available to define the location of the cortex and error is large. For moderate number of sources (3–10) the algorithm finds the head location to within around 4 mm. For large number of sources (> 15) performance becomes constrained by the deterministic (MSP) stage, which fails to recover all the sources.

The effect of SNR on fiducial and co-registration error is shown in Fig. 7(b). At extremely low SNR the head location is known only to within the prior uncertainty (in this case 20 mm). Above *SNR* = 0 we found only a moderate dependence on noise and the algorithm maintained an average fiducial localisation error of approximately 4 mm in all situations except for the noiseless case. Due to the fact that MSP uses discrete patches, there is some quantisation error here, consequently one achieves close to perfect source localisation for moderate fiducial errors (as the nearest incorrect source lies approximately 10 mm away).

One advantage of this approach is the possibility to determine a confidence interval for each source, or fiducial location. Alternatively it is possible to make inference on other indirect metrics such as the location of the global maximum in the cortex. Fig. 7(c) shows this posterior distribution (which through quantization effects, mentioned above, turns out to be one of three possible patch locations) for the single source problem. In this case the fiducials could only be located to within 6.33 mm of their true position. By using BMA (Section Bayesian model averaging) to examine the location of the image maximum over the second half of samples generated by the Metropolis algorithm, and after applying an Occam's window, it was possible to construct an approximate posterior distribution reflecting this uncertainty.

### Experimental data validation

The methodology proposed in this work was tested with previously published data. A detailed description of the experimental set-up and previous data analysis are presented in (Sedley et al., 2012). In brief, auditory evoked responses using a passive listening paradigm and regular interval noise (RIN) pitch-evoking stimuli were fit with bilateral dipolar sources over 13 healthy subjects. We used the data of a single subject from this study but gave the algorithm no information on the subject's head location, i.e. flat priors on location (within the *σ* = 20 mm MEG helmet) and orientation 360° (around all axes). The Metropolis algorithm was performed over approximately 350 iterations per chain in four chains. Unlike the synthetic data examples, with real data some chains generated anomalous samples which were then discarded based on their lower Free energy values.

**Fig. 8:**
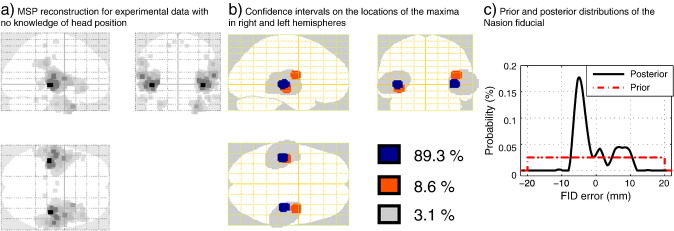
Analysis of single subject auditory evoked response data with no knowledge of head location. (a) Posterior current density estimate. (b) Confidence intervals on the left and right hemisphere maxima (coloured). The grey ellipsoids indicate the 95% confidence intervals on dipole locations for the subject group. (c) Prior and posterior distributions of the Nasion fiducial (based on MEG data and uniform priors); zero shows the nasion estimate based on co-registation.

Fig. 8(a) shows the MSP reconstruction using the fiducial coordinates obtained after performing the Metropolis and BMA estimates. That is, even with no knowledge of head location, the reconstructed current distribution falls within auditory cortex. The problem here is that we do not know the ground truth (either in terms of head location or current distribution). However, we can compare the confidence interval on the maximum, for this individual, against the confidence interval obtained from the group study. Fig. 8(b) shows the 95% confidence ellipsoid based on the dipole fit results of (Sedley et al., 2012) in grey. Overlaid in colour are the confidence intervals on the locations of the left and right hemisphere image maxima based on the single subject's data and without knowledge of head position.

Fig. 8(c) shows the prior and posterior distributions of the Nasion fiducial location. Zero indicates the location of the fiducial location estimated through co-registration. That is, here (in the absence of any other prior information) the MEG data suggest a co-registration error of approximately 7 mm.

## Summary and discussion

We have described a method for mapping brain activity that allows one to account for uncertainty in anatomical parameters which non-linearly affect the MEG forward problem. In order to illustrate this approach we have presented a robust method of MEG source reconstruction that requires only approximate prior knowledge of head location to produce accurate estimates of current density.

We have shown that it is possible to estimate where the brain is to within 4 mm (see Section Simulation results) based purely on MEG data with a signal to noise ratio of zero. Importantly this method provides posterior distributions on current density (or source location), that properly account for not only measurement but also co-registration noise.

Our approach combined deterministic and stochastic Bayesian inference procedures. For a given head location we used the MSP algorithm to estimate sources, and to provide an estimate of the model evidence. A Metropolis search was then used to generate a posterior distribution over possible head locations, and finally the models were combined using BMA. The model averaging stage is simple and increases the robustness of the solution. We stress that the method is used to illustrate what is possible, but its successful empirical realisation depends on knowledge of other unknowns (in this case the correct patch extents and centres) which reflect the true underlying current distribution. Here we have assumed that these other parameters are known, but (given more accurate knowledge of where the brain is) they could be estimated using the same approach. That said, we were encouraged to find that we were still able to provide plausible estimates of the cortical current distribution for real data with no prior knowledge of head position (Fig. 8).

To avoid local maxima in the posterior distribution of the head location a Metropolis search algorithm was implemented. The Metropolis search showed fast and accurate convergence for a MCMC technique. Here, for the purposes of illustration, we used a flat prior on the head location *p(h)* however, in application, one will generally have some information on the true head location (for example, a Gaussian with standard deviation of 5 mm centred around the measured fiducial locations) which will improve the accuracy of the current estimates and also decrease the computation time.

The BMA step is able to pool estimates from across a range of optimisation steps and weight them by their model evidence. This gives a degree of robustness to the process (see Fig. 4(c)) and importantly provides us with useful posterior estimates of not only the head location but also the estimate of current distribution, or alternatively spatial confidence bounds on the locations of local maxima. That is, even if one were not interested in the precise location of the brain (to within 10 mm say); one can still properly account for co-registration errors on the current density estimation.

We were initially surprised to see that no matter how high the SNR was, we were not able to perfectly recover the fiducial locations. It seems that this is a constraint due to the current implementation of the MSP algorithm in which cortical activity is modelled by discrete, non-overlapping patches; this gives rise to a certain level of quantisation error which means that fiducial localisation error can be non-zero for zero source localisation error (see Fig. 7(b)). We also should emphasize that the framework proposed here is not explicitly linked to the MSP algorithm but can sit around any inversion algorithm that provides some form of model evidence.

Continuing on this note, we found that the framework was constrained by the determinsitic inversion stage (the MSP) for large (> 15) numbers of sources (due to the increasingly complex optimisation problem) whereas for small numbers (< 3) of sources, although the MSP worked perfectly, the location of the cortex was poorly defined; there presumably being many positions and orientations of the head that a single source would fit and explain the data equally well.

In this work we have tackled the problem of locating the cortical surface. It should be noted that this general form (MCMC followed by BMA) is a robust method which could be applied to many problems in M/EEG. For example, the estimate of spatial extent of MSP patches, the estimation of conductivity parameters in EEG or as a method to robustly combine algorithms with different prior assumptions (Trujillo-Barreto et al., 2004).

An alternative way to look at the method is to view it as a way of testing the validity of the assumptions behind a source reconstruction algorithm and the forward model. That is, if we had made a nonsensical source reconstruction then we would not have been able to recover where the brain was. Indeed, using LORETA-like priors we were unable to find the location of the electrical activity we had simulated using the same smoother that generates the MSP priors (Green's function, see (Harrison et al., 2007)). That is, in reality, where we do not know what the appropriate functional priors are, but we do know approximately where the head is, we have a method to judge between inversion schemes. Similarly, the higher the spatial resolution/robustness of the algorithm the tighter the confidence bounds on where the brain is. For example (Hillebrand & Barnes, 2003) found that errors in cortical location had little effect on minimum norm as opposed to beamformer solutions. One could also use the approach to test between forward models (canonical vs. individual meshes, for example (Henson et al., 2009)); the advantage being that one could take full account of co-registration errors which might mask the improvement in model evidence given by a specific forward model.

An important application of our method, if not to find out where the brain is, is that one can put a posterior distribution on the locations of the source space maxima which accounts for noise in the measurement of the data and noise in the estimate of head location. Such distributions allow one to make spatial inference on the location of a source, quantify how likely it is to sit in a certain cortical area or be spatially distinguished from another source.

## Footnotes

1 http://www.fil.ion.ucl.ac.uk/spm/
