# Corrigendum to “A general Bayesian treatment for MEG source reconstruction incorporating lead field uncertainty” [Neuroimge 60/2 (2012) 1194–1204]

**DOI:** 10.1016/j.neuroimage.2012.09.013

**Published:** 2013-05-15

**Authors:** J.D. López, W.D. Penny, J.J. Espinosa, G.R. Barnes

**Affiliations:** a Mechatronics School, Bl. M8-108 Facultad de Minas, Universidad Nacional de Colombia, Medellín, Colombia; b Wellcome Trust Centre for Neuroimaging, University College London, WC1N 3BG, London, UK

The authors regret that the following was not correct in the original manuscript:

1. *Page 1194*: Update affiliation *b* with:
  Wellcome Trust Centre for Neuroimaging, University College London, WC1N 3BG, London, UK.
2. *Page 1195*: Separate both probability distributions:
  $$\begin{array}{cc} \;p\left(J\right)=N\left(J;0,Q\right) & p\left(\epsilon \right)=N\left(\epsilon ;0,R\right) \end{array}\text{.}$$
3. *Page 1196*: Separate both probability distributions:
  $$\begin{array}{cc} p\left(\lambda \right)=N\left(\lambda ;\nu ,C_{\lambda }\right) & q\left(\lambda \right)=N\left(\lambda ;\mu {\text{,Σ}}_{\lambda }\right) \end{array}\text{.}$$
4. Figure 8b: Replace with the following figure:

**Figure:**
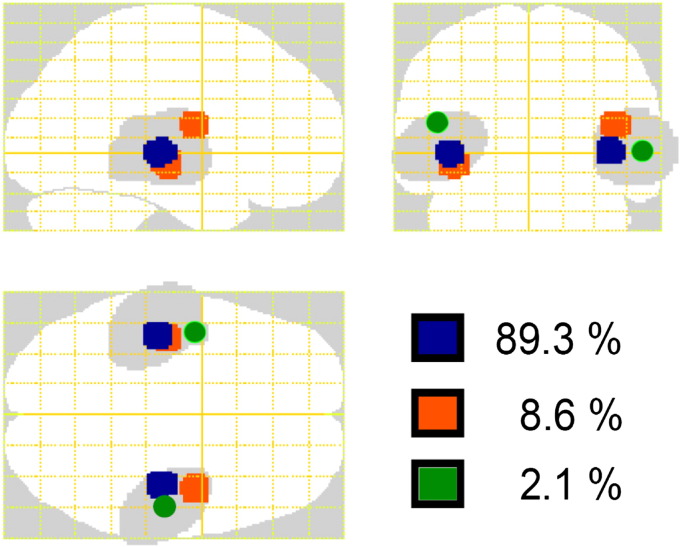

The authors would like to apologize for any inconvenience caused.

